# Laboratory and field evaluation of a low-cost methane sensor and key environmental factors for sensor calibration†

**DOI:** 10.1039/d2ea00100d

**Published:** 2023-02-21

**Authors:** Joyce J. Y. Lin, Colby Buehler, Abhirup Datta, Drew R. Gentner, Kirsten Koehler, Misti Levy Zamora

**Affiliations:** a Johns Hopkins University Bloomberg School of Public Health, Environmental Health and Engineering Baltimore MD 21205-2103 USA; b SEARCH (Solutions for Energy, Air, Climate and Health) Center, Yale University New Haven CT 06520 USA; c Chemical and Environmental Engineering, Yale University New Haven CT 06520 USA; d Johns Hopkins University Bloomberg School of Public Health, Department of Biostatistics Baltimore MD 21205-2103 USA; e Department of Public Health Sciences, UConn School of Medicine, University of Connecticut Health Center Farmington CT USA 06032-1941 mzamora@uchc.edu

## Abstract

Low-cost sensors enable finer-scale spatiotemporal measurements within the existing methane (CH_4_) monitoring infrastructure and could help cities mitigate CH_4_ emissions to meet their climate goals. While initial studies of low-cost CH_4_ sensors have shown potential for effective CH_4_ measurement at ambient concentrations, sensor deployment remains limited due to questions about interferences and calibration across environments and seasons. This study evaluates sensor performance across seasons with specific attention paid to the sensor's understudied carbon monoxide (CO) interferences and environmental dependencies through long-term ambient co-location in an urban environment. The sensor was first evaluated in a laboratory using chamber calibration and co-location experiments, and then in the field through two 8 week co-locations with a reference CH_4_ instrument. In the laboratory, the sensor was sensitive to CH_4_ concentrations below ambient background concentrations. Different sensor units responded similarly to changing CH_4_, CO, temperature, and humidity conditions but required individual calibrations to account for differences in sensor response factors. When deployed in-field, co-located with a reference instrument near Baltimore, MD, the sensor captured diurnal trends in hourly CH_4_ concentration after corrections for temperature, absolute humidity, CO concentration, and hour of day. Variable performance was observed across seasons with the sensor performing well (*R*^2^ = 0.65; percent bias 3.12%; RMSE 0.10 ppm) in the winter validation period and less accurately (*R*^2^ = 0.12; percent bias 3.01%; RMSE 0.08 ppm) in the summer validation period where there was less dynamic range in CH_4_ concentrations. The results highlight the utility of sensor deployment in more variable ambient CH_4_ conditions and demonstrate the importance of accounting for temperature and humidity dependencies as well as co-located CO concentrations with low-cost CH_4_ measurements. We show this can be addressed *via* Multiple Linear Regression (MLR) models accounting for key covariates to enable urban measurements in areas with CH_4_ enhancement. Together with individualized calibration prior to deployment, the sensor shows promise for use in low-cost sensor networks and represents a valuable supplement to existing monitoring strategies to identify CH_4_ hotspots.

## Abstract 2

Low-cost electrochemical methane sensor shows improved measurement accuracy after corrections for carbon monoxide, absolute humidity, temperature, and adjusting for time of day in an urban environment.

## Graphical abstract

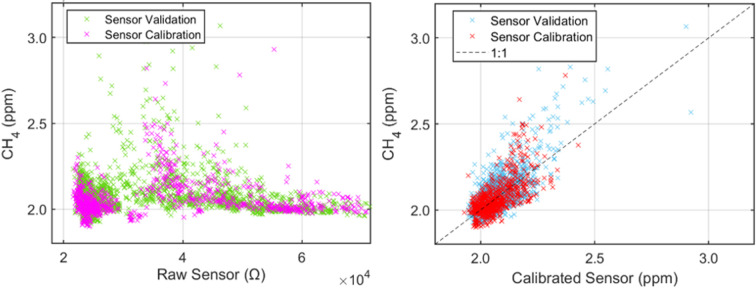

**Environmental significance**

This study evaluates the utility of a low-cost electrochemical sensor for methane (CH_4_) monitoring across seasons in the mid-Atlantic environment. Even as low-cost sensors have risen in popularity for the measurement of other pollutants (PM, CO, NO_2_), their deployment is still limited for the measurement of CH_4_ due to uncertainties about sensor cross-sensitivities and performance across seasons. Here we provide laboratory and field evaluations of the Figaro TGS2600 sensor to identify key covariates for calibration and recommendations for deployment in complex urban environments. A novel correction for the sensor's known CO cross-sensitivity is provided using a low-cost CO sensor that is deployed alongside the TGS2600 CH_4_ sensor in the configuration of a multi-pollutant sensor box.

## 1. Introduction

Methane (CH_4_) plays an important role in the trajectory of global climate change and associated health effects. CH_4_ is around 25 times more effective than carbon dioxide (CO_2_) at trapping heat in the atmosphere and contributes at least a quarter of gross global warming under current emission conditions.^1,2^ In addition to its strong warming potential, CH_4_ is known to increase ground-level ozone over larger spatial scales, and ozone is a major contributor to air pollution-related mortality in the Northern Hemisphere.^3^ CH_4_ is released into the atmosphere through both natural (*e.g.*, wetlands, volcanic releases, wildfires) and anthropogenic sources (*e.g.*, oil and natural gas extraction, landfills, agricultural sector), but anthropogenic CH_4_ emissions account for more than 50% of the global CH_4_ budget.^4,5^ As a result, urban areas have become focal points of CH_4_ monitoring and key intervention points for CH_4_ emission mitigation.

The abundant interest in CH_4_ mitigation has led to numerous direct and indirect efforts to identify CH_4_ sources and sinks. Direct methods such as tower, aircraft, and vehicle measurement campaigns have often been used to monitor local CH_4_ emissions and trends over time.^6,7^ Meanwhile, indirect estimation methods using industry-reported emission inventories have traditionally been used to characterize national and regional CH_4_ emissions in the US.^8^ Yet, there remain large discrepancies across CH_4_ emission estimates and each of these efforts alone cannot answer lingering questions about CH_4_ sources.^9–12^ According to a 2013 study by Miller *et al.*, CH_4_ emissions in the US Environmental Protection Agency (EPA) inventories were underestimated by up to a factor of two and attributed to the likely under-characterization of local CH_4_ leakages.^13^ While direct measurement strategies are best suited to fill this gap, established direct measurement methods often have geospatial limitations that prevent the measurement of CH_4_ with the spatiotemporal resolution needed to assist with source identification in complex urban environments. Tower measurement strategies typically rely on rigorously tested spectroscopic instruments which while highly precise and accurate, can cost upwards of $100 000. As such, they tend to be operated at sparsely located stationary monitoring centers as part of regional or national monitoring efforts. Aircraft and vehicle measurement campaigns benefit from mobile sampling and can provide a snapshot of spatial CH_4_ variability in real-time. However, they too are geospatially limited by their frequency and routes. Given these limitations in the existing CH_4_ monitoring infrastructure, there is a need for additional CH_4_ measurement strategies with higher spatial and temporal resolution to help cities meet their climate goals.

Low-cost sensors have gained popularity in recent years due to their reduced price points, smaller size, and lower power requirements. These sensors are scalable and uniquely suited for deployment in high-density networks alongside other low-cost sensors for multipollutant monitoring in both static and mobile configurations. While there is wide-scale adoption for the measurement of other pollutants like particulate matter, carbon monoxide (CO), and nitrogen dioxide,^14–17^ low-cost sensors are not yet widely implemented for the measurement of CH_4_. The scientific literature for low-cost CH_4_ sensor calibration for deployment in ambient conditions is limited and it remains unclear whether measurements *via* these sensors are adequate for research and monitoring purposes. Additional evaluation of the sensor's response to other pollutants and environmental conditions is needed to determine the suitability of low-cost CH_4_ sensors to supplement existing CH_4_ monitoring efforts.

The Figaro Taguchi Gas Sensor TGS2600 (Figaro USA Inc., Arlington Heights, Illinois) hereafter referred to as the “sensor” or “TGS2600 sensor”, is a low-cost metal oxide sensor that has shown promising results for CH_4_ monitoring at ambient concentrations. Since Eugster and Kling (2012) first identified the ability of this sensor to observe diurnal CH_4_ trends in low arctic conditions in Alaska, the sensor has been evaluated for identifying fugitive CH_4_ emissions from natural gas terminals in the UK,^18^ measuring CH_4_ emissions at sites proximal to oil and gas development in Denver, Colorado,^19^ and for community-based research in urban Los Angeles, California.^19^ In 2019, Eugster *et al.* also presented a long-term evaluation of the sensor which estimated the sensor lifespan to be around 10–13 years providing further support for its use in long-term monitoring networks.^20^ A similar sensor from the same manufacturer (Figaro 2611-E00), which includes a built-in filter to remove interference from non-methane oxidizable gases, has also reported efficacy for CH_4_ monitoring after correction for the sensor's temperature and relative humidity (RH) dependencies.^21^

The sensor's temperature and humidity dependencies have been characterized in both laboratory and field settings, but few studies have had the capability to correct for potential cross-sensitivities to other pollutants. Despite the manufacturer-indicated cross-sensitivity to CO and other volatile organic compounds (VOCs), only one 2018 study by Collier-Oxandale *et al.* evaluated CO sensitivity using reference CO concentrations from a nearby monitoring tower.^19^ Cross-sensitivity to CO was observed in both Denver and Los Angeles with the differences in the variance explained by CO between deployments.^19^ However, since higher CO concentrations generally occur in areas proximal to traffic or other combustion processes, adjustments made to the sensor using a regional, but not co-located, CO reference instrument may not accurately reflect the CO concentrations experienced by the sensor. Furthermore, the previous deployments in Denver and Los Angeles occurred in the summer seasons during which CO concentrations tend to be much lower than in the winter months, a seasonal pattern is also present in the mid-Atlantic region of the US.^22^ Since sensor calibration, accuracy, and susceptibility to CO cross-sensitivity is likely to differ by season and by ambient CO concentration, further evaluation of the sensor's cross-sensitivity to CO with a co-located low-cost sensor is needed to understand the suitability of the sensor's widespread deployment in large-scale, high-density monitoring networks across the US.

This study aims to evaluate the suitability of the Figaro TGS 2600 gas sensor for ambient CH_4_ monitoring across seasons in an urban Mid-Atlantic environment with specific attention to the sensor's CO cross-sensitivity and other environmental factors. This work expands on existing evidence supporting the sensor's use in complex urban settings and provides the basis for its long-term deployment in a citywide multipollutant sensor network in Baltimore, MD. To our knowledge, this is one of the first studies to deploy a CO sensor alongside the TGS 2600 gas sensor in the configuration of a multi-pollutant sensor box which allows us to present more accurate CO corrections than previously available.

## 2. Methods

### 2.1 Sensor incorporation and monitor design

The Solutions for Energy, Air, Climate, and Health (SEARCH) center designed custom multipollutant monitors with the low-cost Figaro Taguchi Gas Sensor TGS2600 built in to measure CH_4_. This sensor is operated using tailored electronics with low-noise circuitry/power alongside a suite of other low-cost sensors to measure the concentrations of CO, NO_2_, NO, CO_2_, O_3_, and size-resolved particulate matter (PM).^23^ The multipollutant monitor also incorporates a Sensirion SHT25 digital temperature and humidity sensor (Sensirion, Staefa, Switzerland), which enables the assessment of sensor dependence on humidity and temperature. Details of how the various sensors are configured and operated in the multipollutant monitor are provided in detail by Buehler *et al.*^23^ There are 45 multipollutant monitors installed in a long-term, citywide low-cost sensing network in Baltimore, MD.

According to the manufacturer, the TGS2600 sensor reports a drop in resistance in the presence of a de-oxidizing gas. The sensor specifications sheet also indicates sensitivities to CO, isobutane, ethanol, and hydrogen.^24^ To reduce VOC interferences in the sensing component, the sensors used in this study were covered with a layer of activated carbon-impregnated cloth (Zorflex® Double Weave) held in place with a retaining ring that was wrapped on the sides with Teflon tape (see ref. 23). Under ambient conditions, the activated carbon in the cloth substrate does not interact with CH_4_ or CO and potential interactions with water vapor would be weak. The lifetime of this method is dependent on ambient VOC concentrations, but this technique was previously shown to be effective in filtering out ethanol interferences as high as 2% in a controlled laboratory setting and remained effective after continuous outdoor VOC exposure for 3 months.^23^ With this new multipollutant deployment setup, we seek to control for sensor cross-sensitivities to other pollutants and specifically provide an additional correction for the sensor's known cross-sensitivities to CO using an on-board Alphasense CO-A4 sensor (Alphasense, UK).

### 2.2 Laboratory experiments

Chamber and in-room tests were performed in the laboratory to evaluate sensor performance. The sensor reports in resistance units and requires a calibration factor specific to each sensor to convert the raw resistance data to concentration units. Since the laboratory experiments aim to answer questions about inter-sensor comparability and responses to environmental conditions, the sensor output is compared in its original resistance units for these tests.

#### 2.2.1 Chamber experiments

In the laboratory at the Johns Hopkins Bloomberg School of Public Health, sensors were evaluated for cross-sensitivities to CO, CO_2_, NO, and NO_2_, as well as temperature and humidity dependencies in controlled chamber experiments prior to their deployment in the field. Two multipollutant monitors with embedded TGS2600 sensors were operated inside a custom-built steel chamber (0.71 m × 1.35 m × 0.89 m), equipped with a filtered air inlet, vacuum exhaust, two internal fans, and three sampling ports. The sensor responses to CH_4_ (0–3 ppm), CO (0–4 ppm), CO_2_ (400–700 ppm), NO (0–0.05 ppm), NO_2_ (0–0.05 ppm), temperature (15–38 °C), and relative humidity (13–62%) were determined at 1 min averaging times. For these experiments, each gas was introduced to the chamber through filtered air inlets, diluted to the specified concentration, and held for 60 minutes at each concentration. The temperature was controlled by introducing heat or ice packs to the calibration chamber. The humidity was controlled using an ultrasonic DP 100 medical nebulizer with deionized water to increase water vapor in the chamber. To better characterize the sensor's response to humidity independent of temperature, relative humidity measurements reported by the digital humidity sensor were converted to absolute humidity (AH) in g m^−3^ using eqn (1) with measurements from the multi-pollutant monitor where *T* is the temperature in degrees Celsius and RH is relative humidity expressed in percent.^25^1
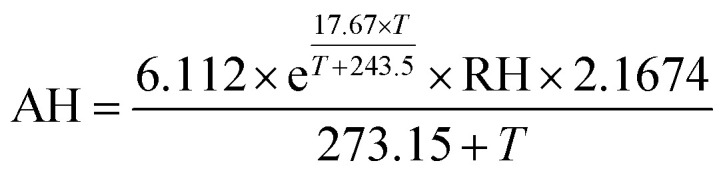


#### 2.2.2 Laboratory room co-location

Outside of the calibration chamber, 9 multipollutant monitors were co-located in a laboratory setting to assess inter-sensor variability over an 8 day period. Monitors were clustered on a laboratory bench and subjected to typical indoor temperature and RH changes in the laboratory room (controlled with an HVAC system) over the co-location period with no notable activity occurring in the room during this time.

### 2.3 In-field testing

The in-field tests aim to compare the sensor to a reference instrument measuring ambient CH_4_ concentrations (*i.e.*, ppm). One multipollutant monitor was co-located with a Picarro Cavity Ring-Down Spectrometry (CRDS) Analyzer at the National Institute of Standards and Technology (NIST) Northeast Corridor Urban Test Bed project's HAL tower site in Halethorpe, MD.^26^ This reference CRDS instrument is operated in an enclosed indoor monitoring space that is connected to a sampling inlet located 29 m above ground on a communications tower. The co-located multipollutant sensor box was also set up in the enclosed space with airflow from the 29 m sampling inlet. The instruments were co-located from January to September 2020 but the sensor data from March to July were inaccessible due to COVID-19 restrictions at the sampling site. As a result, co-location data presented from this study are for 8 weeks in the winter (January–March 2020) and 8 weeks in the summer (July–September 2020). The first two weeks from the winter and summer datasets were used as calibration periods so that the models could be trained using data across seasons. The remaining time (6 weeks winter, and 6 weeks summer) was used for validation. Similar results were observed when using random calibration and validation periods across the total co-location period (not shown). Field co-located sensor data were converted to 1 h averages to correspond with the resolution of the available CRDS reference data.

### 2.4 Data analysis

Multiple linear regressions (MLRs) were used to predict CH_4_ concentration in reference units based on sensor resistance (*R*_s_) and other relevant predictors. A generic MLR model used to calibrate low-cost sensors is given by:

2 Reference_CH_4__(*t*) = *β*_0_ + *β*_1_ × sensor_CH_4__(*t*) + *β*_*n*_ × predictor_*n*_(*t*)

Reference_CH_4__ is the CH_4_ concentration from the CRDS reference instrument at time *t*, *β*_0_ is the constant intercept, *β*_1_ is the coefficient applied to the uncalibrated sensor resistance for CH_4_ at time *t*, and *β*_*n*_ is the coefficient for predictor_*n*_ at time *t* (hourly averages).

Predictors that were considered in the models included internal multi-pollutant monitor measurements of temperature, AH, CO, as well as hour of day, and various combinations of interactions between these predictors. The inclusions of temperature, AH, and CO were based on results from the laboratory evaluation, which indicated sensor responses to these predictors in the calibration chamber. CO_2_, NO, and NO_2_ were not considered since the sensor exhibited no notable responses to these predictors in the calibration chamber. CO concentration from the nearest regional reference CO instrument was also included to evaluate potential improvements to the model using the onboard low-cost CO sensor.

Predictors that exhibited non-linearity in the chamber experiments (temperature and AH) were evaluated in their functional forms from laboratory testing and as piecewise linear responses using a spline at the median value since no inflection points were observed. Interaction terms between the sensor and AH, the sensor and temperature as well as temperature and AH were evaluated based on existing knowledge of the Figaro TGS2600 sensor's dependencies on temperature and humidity, the Alphasense CO-A4 sensor's known dependency on temperature, and the interaction between temperature and humidity in ambient environments.

To account for diurnal trends in CH_4_ concentration, a time-specific predictor for the hour of day was included as a 23-level categorical variable corresponding to the hours of the day from 1–23 with hour 0 as the baseline. Since the field co-location took place for less than one full year, it was not possible to distinguish whether the changes over time were attributable to seasonal variability over the co-location period or instrument drift. As a result, drift was not included in the final calibration models presented in this study.

The performance of the models was assessed with several metrics. The percent bias was calculated as:3
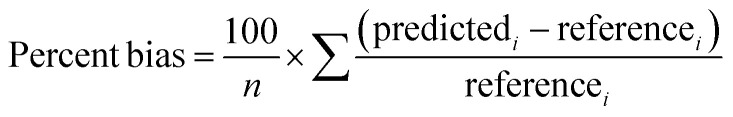


The percent bias was determined across all 1 h averaged concentrations recorded during the deployment. The predicted_*i*_ corresponds to the calibrated *i*-th hourly averaged sensor CH_4_ concentration from the training or evaluation period. Reference_*i*_ corresponds to the *i*-th hourly averaged CH_4_ concentration from the reference instrument.

Model selection was based on the coefficient of determination (*R*^2^) and the root mean squared error (RMSE). In cases where the *R*^2^ and RMSE were comparable across models, the model with the smallest Bayesian Information Criterion (BIC) was selected as the final model. Since the BIC penalizes based on the number of parameters in the model, more parsimonious models with lower values are preferred and may help limit overfitting the model to our data. Analyses were performed using Matlab R2019b.

## 3. Results and discussion

### 3.1 Laboratory results

**Fig. 1:**
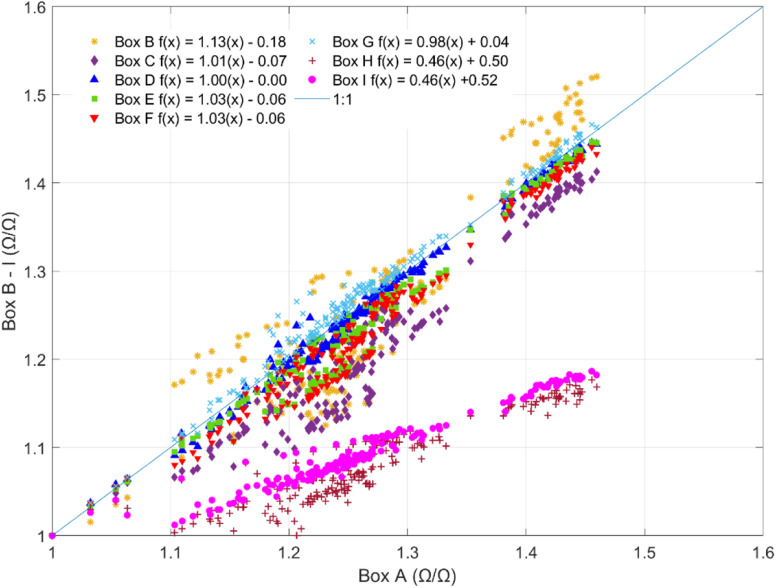
Inter-sensor slope comparisons for 9 sensor boxes co-located in the laboratory over 8 consecutive days at 1 h resolution compared to Box A. For the purposes of comparison, sensor responses are normalized by their minimum observed values.

Sensors co-located in the climate-controlled laboratory exhibited similar temporal trends and responses to indoor environmental conditions but reported substantially different resistances. The sensors exhibited strong correlations (*R* = 0.89–1.0) between sensor pairs (ESI Fig. S1†) but responded with varying magnitudes to minor temperature and AH fluctuations in the laboratory. Fig. 1 shows the slope comparisons for 9 sensors (Box B–I) compared to Box A, which were co-located in the laboratory over 8 consecutive days at 1 h resolution. For intercomparison, sensor responses were first normalized by dividing the reported resistances by their minimum observed values at a shared time point to account for baseline resistance differences in the sensors (1.2 × 10^4^ to 4.7 × 10^4^ Ω). The non-baseline normalized inter-sensor comparison is provided in ESI Fig. S2.† The magnitude of response per unit of exposure is comparable for six of the eight sensors (slopes ranging from 0.98–1.13) but two of the sensors had lower slopes at 0.46 (Fig. 1). Given the observed differences in both baseline resistances and sensor slopes, it is recommended that the sensors undergo individual calibration prior to deployment.

**Fig. 2:**
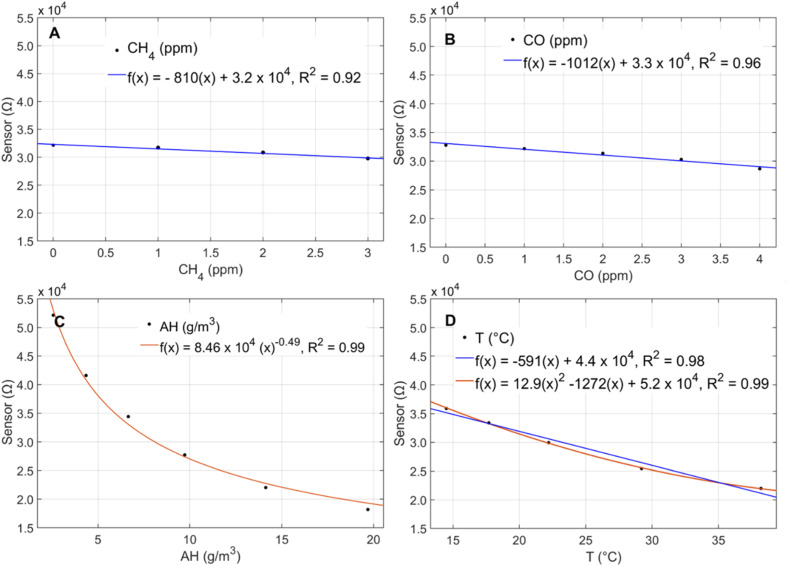
Sensor response to (A) CH_4_, (B) CO, (C) AH, and (D) temperature in calibration chamber experiments.

In the chamber calibration experiments, the sensors exhibited significant inverse responses to CH_4_, AH, temperature, and CO and no notable responses to CO_2_, NO, or NO_2_ (ESI Fig. S3†). The CO calibration for the onboard low-cost CO sensor is presented in ESI Fig. S4.† Given the differences in the sensors' baseline resistances and response factors, data from only one sensor is shown to visualize the general functional forms of sensor response to the significant predictors (Fig. 2A–D).

The pollutant concentrations tested in the chamber were chosen to reflect averages expected in ambient environments in the United States. The temperature and humidity were modified to the extent possible using the calibration chamber setup, but the temperatures tested did not reach the full range experienced in the ambient Mid-Atlantic environment, which ranges from −4 °C to 37 °C throughout a typical year.^22^ Nonetheless, the sensors exhibited significant non-linear responses to temperature over 15 °C–38 °C and AH over 3 g m^−3^–14 g m^−3^ (Fig. 2C and D), consistent with the manufacturer's indicated sensitivities to these predictors.^24^ The manufacturer also indicates that the sensor exhibits non-linear responses to CH_4_ and CO over concentrations ranging over 0–100 ppm; however, we evaluated these predictors over a smaller range with concentrations more applicable to ambient conditions. We observed linear sensor responses to CH_4_ over 0–3 ppm (Fig. 2A) and CO over 0–4 ppm (Fig. 2B), a trend that was consistent with manufacturer testing within this range.^24^ The sensor's CH_4_ detection limit was assessed in a previous paper detailing the setup of the multipollutant sensor box and found to be below ambient background concentrations.^23^ In the laboratory calibration part of this study, we observed sensor responses at CH_4_ concentrations as low as 0.7 ppm, which is significantly lower than ambient background CH_4_ concentrations reported in environmental studies which range from 1.6–1.9 ppm.^18–20^ Temperature and AH were held constant at approximately 30 °C and 6 g m^−3^, respectively, during the pollutant tests (CH_4_, CO, CO_2_, NO, NO_2_). Throughout the temperature and humidity calibrations, CH_4_ and CO concentrations were held at 2.5 ppm and 1 ppm, respectively. AH remained around 6 g m^−3^ over the course of the temperature calibration while temperatures ranged from 24 °C–30 °C over the course of the humidity calibration. Given the sensor's strong non-linear response to humidity, a 6 °C change in temperature is not expected to affect the direction or functional form of the sensor's response to AH.

### 3.2 In-field co-location results

#### 3.2.1 Uncalibrated observations

**Fig. 3:**
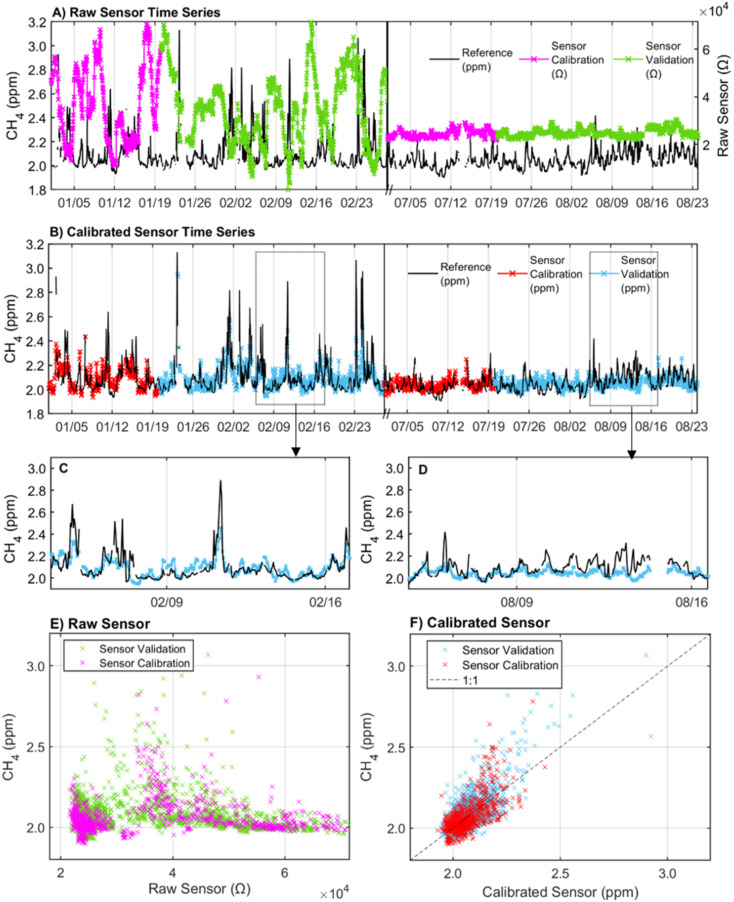
(A) Time series of reference CH_4_ (black) and raw sensor resistances during calibration (pink) and validation (green) co-location periods. (B) Time series of reference and calibrated sensor values split by season and calibration (red) and validation (blue) periods. (C) Subset of the validation period in the winter during a typical week. (D) Subset of the validation period in the summer with poor performance. (E) Scatterplot of raw sensor resistance and reference concentration shown for calibration and validation periods. (F) Scatterplot of calibrated sensor and reference shown separately for calibration and validation periods. Note: the calibrated sensor shown is corrected using Model 5 which included predictors *R*_s_, AH, temperature, CO, and hour of day. Plots (A)–(F) show data at 1 h resolution.

The time series of the 1 h averaged raw sensor data split by calibration (pink) and validation (green) period is shown in Fig. 3A. It is possible to see an inverse relationship between the raw sensor and reference concentrations in the winter months when CH_4_ concentrations ranged from 1.9–3.2 ppm, but the limited variability of CH_4_ concentrations on the 29 m tower in the summer (1.9–2.5 ppm) obscures any further interpretation of the raw data. The scatterplot of the reference concentrations with the raw sensor resistances for the total deployment period (winter and summer combined) split by calibration and validation period, shows two discernible clusters corresponding to the winter and summer seasons but no relationship (*R*^2^ = 0.00) between the raw sensor values and reference CH_4_ concentrations (Fig. 3E). Given the low CH_4_ variance conditions observed in the summer season, focused attention is given to model fit for the winter season during model evaluation.

#### 3.2.2 Model evaluation and selection

Calibration models tested included predictors that the sensor responded to in the laboratory chamber experiments (*i.e.*, CH_4_, AH, temperature, CO). A model with sensor response (*R*_s_) as a linear predictor was used as the base model, which represented only a unit translation and no adjustments for sensor cross-sensitivity or environmental dependency. Next, AH was introduced with a linear predictor and then as a square root (*x*^0.5^) and spline term corresponding its non-linear form observed in the laboratory experiments. Temperature was also added and evaluated as linear, square, and spline terms corresponding to the sensor's functional response to this predictor in the laboratory. Subsequent models tested a linear predictor for CO, a predictor for hour of day as well as interaction terms between the sensor resistance and AH, the sensor resistance and temperature, CO and temperature, and AH and temperature.

Four calibration models with the most significant stepwise increases to model fit are shown in Table 1. A full description of all the models tested in order of increasing complexity is provided in ESI Table S1† with summaries of the model fits by season provided in ESI Table S2a and b.† Models that include CO concentration use the on-board Alphasense CO-A4 sensor that is embedded into the configuration of the co-located multipollutant sensor box. The raw CO values were first corrected for their strong temperature and modest relative humidity dependencies as described by Levy-Zamora *et al.* 2022, before being included as a predictor in the calibration models.^27^

**Table:** Abridged summary of calibration models^a^

Model	Model equation
0	[CH_4_] = *β*_0_ + *β*_1_(*R*_s_)
2	[CH_4_] = *β*_0_ + *β*_1_(*R*_s_) + *β*_2_(AH) + *β*_3_(T)
3	[CH_4_] = *β*_0_ + *β*_1_(*R*_s_) + *β*_2_(AH) + *β*_3_(T) + *β*_4_(CO)
5	[CH_4_] = *β*_0_ + *β*_1_(*R*_s_) + *β*_2_(AH) + *β*_3_(T) + *β*_4_(CO) + *β*_5_(HOD)

a *R*_s_ = resistance (Ω) of sensor in the presence of methane. AH = absolute humidity. *T* = temperature. HOD = hour of day. The full list of models tested is available in the ESI.

Due to the enclosed nature of the NIST measurement station and the constant internal heat generated by the CH_4_ sensor and other sensing components within the multipollutant monitor, the instrument experienced smaller seasonal temperature and AH variations than were observed by temperature and humidity sensors located outdoors. Over the 8 month deployment, the sensor experienced AH ranging from 3 g m^−3^–24 g m^−3^, and temperatures from 17 °C–38 °C. For comparison, the outdoor AH and temperature recorded during this time ranged from 3 g m^−3^–27 g m^−3^ and −7 °C–38 °C, respectively.^22^ As such, there is less range in the deployment data used to generate coefficients for the temperature and humidity predictors. This could lead to greater uncertainty on these predictors at high humidity and low temperature conditions if the box were set up fully outdoors. Nonetheless, the clear evidence of the sensor's temperature and humidity dependencies in the laboratory experiments agrees with previous evaluations of the sensor lending to their inclusions in the final calibration model regardless of significance in the model.

Model fit summaries for the sensor data at 1 h resolution across the entire deployment period are shown in Table 2a and for the data split by winter and summer season in Table 2b. Model 0, using sensor resistance (*R*_s_) as the only predictor, resulted in an overall *R*^2^ of 0 with an RMSE of 0.13 ppm and 3.52% bias in the validation period across the total deployment period (Table 2a). However, when the model is evaluated by winter and summer seasons separately, it is evident that the summer season is the predominant driver of the poor overall *R*^2^. When Model 0 was applied to the winter deployment data only, it resulted in an *R*^2^ of 0.23 with an RMSE of 0.14 ppm and 3.9% bias in the validation period suggesting that the sensor performs better in the more variable CH_4_ variable winter season (Table 2b). Discussions of subsequent models focus on model fit in the winter season during which sensor performance is evidently stronger.

(a) Model fit results for 1 h resolution data over total deployment period. (b) Model fit results for 1 h resolution data split by deployment season

**Table:** 

Model	Calibration			Validation		
	Bias (%)	RMSE (ppm)	*R* ^2^	Bias (%)	RMSE (ppm)	*R* ^2^
0	3.59	0.12	0.00	3.52	0.13	0.01
2	3.11	0.10	0.22	3.73	0.11	0.21
3	2.68	0.09	0.37	2.82	0.08	0.52
5	2.61	0.08	0.43	2.69	0.08	0.55

**Table:** 

Model	Winter						Summer					
	Calibration			Validation			Calibration			Validation		
	Bias (%)	RMSE (ppm)	*R* ^2^	Bias (%)	RMSE (ppm)	*R* ^2^	Bias (%)	RMSE (ppm)	*R* ^2^	Bias (%)	RMSE (ppm)	*R* ^2^
0	3.82	0.13	0.17	3.90	0.14	0.23	2.16	0.06	0.03	2.88	0.08	0.01
2	3.71	0.12	0.31	5.39	0.14	0.34	2.17	0.06	0.07	2.63	0.07	0.19
3	3.04	0.10	0.42	3.09	0.09	0.65	2.04	0.06	0.19	2.24	0.06	0.33
5	3.08	0.10	0.47	3.12	0.09	0.65	1.83	0.05	0.40	3.02	0.08	0.12

For the winter deployment period, the addition of AH and temperature as linear variables in Model 2 further improved the *R*^2^ to 0.34 and resulted in an RMSE of 0.14 ppm and 5.39% bias in the validation period (Table 2b). Across the total deployment period, the addition of these predictors improved model fit from an *R*^2^ of 0 to 0.21 with an RMSE of 0.11, and a 3.73% bias in the validation period (Table 2a). No significant improvements to model fit were observed when AH and *T* were evaluated in square root and square forms. Thus, AH and *T* were kept as linear terms in the final model despite the observed non-linear sensor responses to these predictors in the laboratory.

The greatest improvement to the model fit was observed with the addition of calibrated CO sensor concentration as a linear term (Model 3), which resulted in an overall *R*^2^ of 0.52, RMSE of 0.08 ppm, and 2.82% bias in the validation period across the entire deployment (Table 2a). The addition of an hour of day predictor further improved the model fit to an overall *R*^2^ of 0.55, RMSE of 0.08 ppm, and 2.69% bias in the validation period (Table 2a). When applying this model to the winter deployment only, it resulted in an *R*^2^ of 0.65, RMSE of 0.09 ppm, and 3.12% bias in the validation period (Table 2b).

The inclusion of an interaction term between AH and temperature slightly improved the validation *R*^2^ from 0.55 to 0.59 across the total deployment period but significantly increased the model BIC. Moreover, the addition of the interaction term did not improve the model fit when applied to the winter season only (Model 8 in ESI Table S2a and b†). As a result, Model 5 with linear terms for sensor resistance, AH, temperature, CO, and hour of day was chosen as the final calibration model. The time series of the sensor calibrated using Model 5 in 1 h resolution is provided in Fig. 3B. The scatterplot of the calibrated sensor split by calibration and validation periods across the entire deployment period shows an *R*^2^ of 0.43 in the calibration period and 0.55 in the validation period (Fig. 3F). The *R*^2^ from the validation period appears to be largely influenced by the strong CH_4_ peaks from 2/10–2/29 that are well predicted by the sensor. This led to a higher *R*^2^ (0.55) in the validation period than the calibration period (*R*^2^ = 0.43). When the calibration and validation periods are reversed with the calibration period spanning 2/10–2/29, the calibration *R*^2^ improves to 0.64 (ESI Table S3†).

The calibrated sensor captured CH_4_ dips and peaks over the co-location period but failed to capture the full range of CH_4_ variability. In the winter when CH_4_ concentrations ranged from 1.9 ppm–3.2 ppm, the calibrated sensor underestimated high concentrations (>2.3 ppm) and overestimated minor spikes in CH_4_ concentration below 2.3 ppm (Fig. 3C). This is consistent with knowledge of potential peak underestimation with mean-reverting methods like regression.^28^ The regression approach was chosen despite this limitation given the importance of calibration accessibility and interpretability to a wide range of low-cost sensor users. In the summer, the CH_4_ concentrations used for training (and evaluation) were more limited (1.9–2.5 ppm) at the elevated 29 m tower height, and all tested calibration models performed consistently worse than in the winter (Table 2b and ESI Table S2c†). The less accurate sensor performance in the low CH_4_ variance summer period highlights the need for sufficient dynamic range in training datasets.^28^

Across the total deployment period, the calibrated sensor trended with the CH_4_ concentrations reported by the reference instrument but exhibited a period of consistent underestimation from 08/09–08/16, failing to fully capture short-duration peaks in concentrations (Fig. 3D). However, no abnormalities in the measured environmental conditions were observed during this period. Temperature ranged from 30.8 °C to 35.7 °C, AH ranged from 10 g m^−3^ to 15 g m^−3^, and CO concentrations ranged from 0.2 to 0.3 ppm. These conditions were consistent with conditions observed on days both before and after the underestimation period suggesting that the poor performance may also be related to factors beyond the measured conditions considered in this analysis. Despite these limitations, at 1 h resolution, 58% of the readings were within 2.5% of the reference instrument (0.05 ppm), 87% were within 5% (0.1 ppm), and 98% were within 10% (0.2 ppm) of the reference values during the total co-location period.

**Fig. 4:**
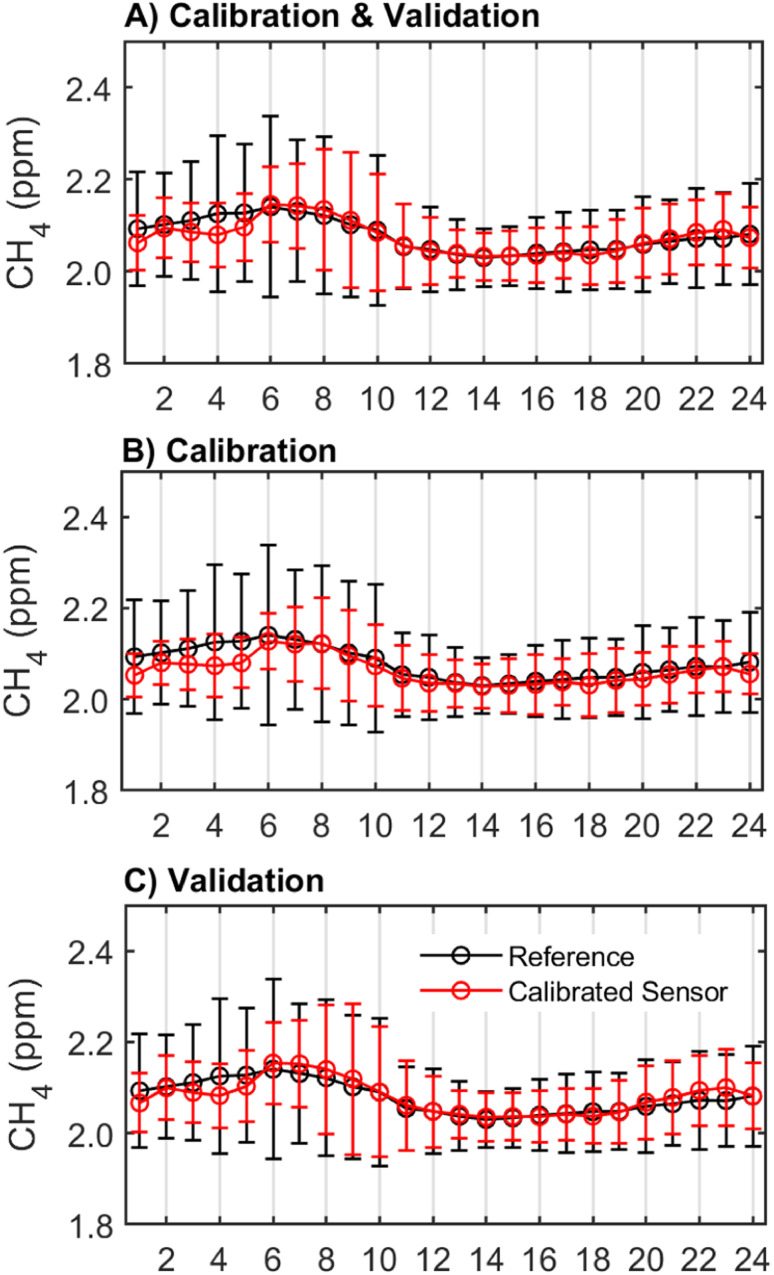
Diurnal patterns in CH_4_ concentrations with arithmetic means (and standard deviations) by the hour of the day for (A) the total deployment period, (B) the calibration period, and (C) the validation period. Note: the data shown is calibrated using Model 5 which included predictors *R*_s_, AH, temperature, CO, and time of day. Plots (A)–(C) show data at 1 h resolution.

One important characteristic of effective CH_4_ monitoring is the identification of diurnal trends or patterns of CH_4_ concentration changes throughout the day. The hourly average concentrations from the sensor followed the same diurnal pattern as the reference instrument but underestimated average CH_4_ concentrations in the early hours between 1 am and 6 am in both the calibration and validation periods (Fig. 4). In the validation period, the sensor also overestimated average CH_4_ concentrations from 6–11 am and 9–11 pm (Fig. 4C). Across seasons and times of day, the sensor reported a smaller range of CH_4_ concentrations than the reference instrument. Between 4 am and 8 am, when there is the greatest variability in CH_4_ concentrations, the standard deviation of the hourly measurements reported by the calibrated sensor is up to 0.3 ppm smaller than that reported by the reference instrument. For example, the calibrated sensor recorded CH_4_ concentration ranges between 1.9 ppm and 2.9 ppm over the co-location period, but the reference instrument recorded CH_4_ concentrations as high as 3.2 ppm during the same period. The inclusion of the hour of day predictor in the calibration model resulted in greater agreement with the reference instrument particularly in the early hours of the morning (6–7 am) and the afternoon (1–8 pm). Importantly, this addition also resulted in a greater agreement between the sensor and reference time series in the low variance summer season (ESI Fig. S5†). This improvement suggests that the hour of day predictor could be accounting for other pollutants or conditions that were not directly measured in this study which exhibit diurnal trends.

A possible contributor to the limited hourly CH_4_ variability during this deployment is the elevated inlet on the measurement tower used for the co-located in-field testing at the NIST Northeast Corridor Urban Test Bed site. The sampling inlets for this project were selected to strike a balance between the identification of anthropogenic greenhouse gas emissions from the surrounding urban area and the ability to simulate observations through transport and dispersion models across regional towers across the Northeast.^26^ However, in denser urban monitoring networks, it is beneficial to measure trace gas concentrations closer to ground level sources so that finer spatial gradients can be used to identify potential sources and emissions estimates.^26^ Since both the reference and low-cost instruments were sampling from an inlet located 29 m above ground level in suburban Baltimore, CH_4_ emissions are likely more diluted compared to at surface level where sensors may experience larger fluctuations due to greater proximity to CH_4_ sources. As such, the sensor may be better suited for ground-level measurement with surface-level co-location to provide a wider range of data to train the sensor calibration models.

Despite the elevated sampling inlet, our findings suggest that after adjusting for temperature and humidity dependencies, filtering for VOC interferences using the activated carbon filter, and correcting for CO cross-sensitivity, the sensor is a valuable supplement to existing monitoring strategies to identify localized trends and CH_4_ hotspots. With an overall 2.8% bias from the reference and 0.08 ppm RMSE throughout the 16 week deployment, the sensor effectively captures diurnal and seasonal CH_4_ trends, with notably better performance in high CH_4_ variance conditions. Thus, in combination with individualized field calibration prior to deployment, the sensor is a strong candidate for deployment as part of low-cost sensing networks to identify CH_4_ emissions trends proximal to potential emissions sources.

#### 3.2.3 Comparison with literature

To contextualize sensor performance from this deployment, we show a summary of our co-location data with performance metrics from several other field deployments in the literature (Table 3). The data presented are specific to their particular deployments; thus, differences in performance and the calibration models used may be attributed to differences in sampling locations, environmental conditions, and testing durations across studies.

**Table:** Comparison of the sensor performance across published field studies^a^

Study	Deployment location	Deployment length	Time resolution	CH_4_ range	Predictors in final model	*R* ^2^
Eugster & Kling, 2012	Low arctic Alaska, ice free summer	10 weeks	1 min	1.8–2.0 ppm	CH_4_ sensor, RH, temperature, time	0.19
Collier-Oxandale *et al.*, 2018	10 × 10 km grid in Colorado (14 sensors)	4 weeks	1 min	1.6–6.7 ppm	CH_4_ sensor, temperature–CH_4_ interaction, AH, time	0.42
	Los Angeles, CA. near oil extraction sites	8 weeks	1 min	1.9–3.4 ppm	CH_4_ sensor, temperature–CH_4_ sensor interaction, AH, time, temperature–AH interaction, hour of day	0.74
Casey *et al.*, 2019	Colorado, oil and gas production area	12 weeks	1 min	1.8–4.4 ppm	CH_4_ sensor, temperature, RH, CO_2_	0.66
Eugster *et al.*, 2019	Low arctic Alaska	6 years	30 min	1.8–2.1 ppm	CH_4_ sensor, temperature, AH, CH_4_ sensor–temperature interaction, CH_4_ sensor–AH interaction, CH_4_ sensor–temperature–AH interaction	0.42
Riddick *et al.*, 2020	United Kingdom, near gas terminal	1 week	1 min	1.9–5.5 ppm	CH_4_ sensor, RH, temperature	0.27
This study^b^	Baltimore, Maryland	16 weeks	1 hour	1.9–3.2 ppm	CH_4_ sensor, AH, temperature, CO, hour of day	0.52

a RH = relative humidity. AH = absolute humidity. Time = time since deployment.

b Using Model 5 with data from the total deployment period across winter and summer seasons.

Studies varied by deployment length, data resolution, and predictors used in the final calibration models. Most studies evaluated sensor performance at 1 min resolution for deployments spanning between 1 and 10 weeks. One long-term (6 year) study by Eugster *et al.* in 2019 reported results integrated over 30 min.^20^ This study was the first cross seasonal deployment of the sensor in urban conditions and evaluated sensor performance at 1 h resolution over 16 weeks (8 weeks in the winter and 8 weeks in the summer) to determine the suitability of the sensor for long-term deployment for the monitoring of spatial and seasonal CH_4_ trends. Our calibration model, along with all published research on this sensor, presented adjustments for humidity and temperature, highlighting the importance of correcting for these sensor dependencies during deployment regardless of deployment length or location.

Overall, we reported the third highest *R*^2^ (0.52) of published research on the sensor following the 2018 study by Collier-Oxandale *et al.*^19^ near oil and gas extraction sites in Los Angeles (*R*^2^ = 0.74), and the 2019 study by Casey *et al.*^29^ in oil and gas production areas in Colorado (*R*^2^ = 0.66). Compared to previous deployments of the sensor in urban settings, this study exposed the sensor to more humid mid-Atlantic conditions with lower and less variable CH_4_ concentrations away from immediate oil and gas production activity. As a result, this study experienced the smallest range of CH_4_ concentrations (1.9–3.2 ppm) of all existing urban studies across the entire deployment period with the summer season experiencing and an even more limited range from 1.9–2.5 ppm. There were notable differences in sensor calibration comparing the winter (*R*^2^ = 0.65) and summer (*R*^2^ = 0.12) seasons with all tested calibration models performing substantially better in the winter deployment period. Given the poor performance observed in the low CH_4_ variability summer season atop the sampling tower, we recommend that future deployments of this sensor should focus on ground-level co-locations that train the sensor in more dynamic CH_4_ concentration conditions to overcome low variance challenges associated with seasonal changes in CH_4_ concentrations.

To our knowledge, this is also the only deployment to date with the capability of filtering out VOC interference and adjusting for point of measurement CO cross-sensitivity. The sensor's sensing component was covered with carbon-impregnated cloth to filter ethanol interferences, and the sensor's known cross-sensitivity to CO was corrected for using an onboard co-located low-cost CO sensor. The unique design of the multipollutant sensor box which included the addition of a VOC filter and measured multiple other environmental conditions and pollutants at the sampling site allowed us to account for all the cross-sensitivities indicated by the manufacturer and present more accurate CO corrections than previously available. Attempts to adjust for the sensor's CO cross-sensitivity using the nearest regional reference CO instrument did not lead to improvements in the calibration model. However, the addition of corrected CO concentration from the onboard low-cost sensor resulted in significant improvements to model *R*^2^ in both winter and summer seasons (Table 2b). This demonstrates the value of using co-located CO measurement, especially in urban environments that are susceptible to CO co-emissions from local vehicular emissions. The significant improvements made with the inclusion of the CO predictor also raise additional questions about urban co-exposures and exposure mixtures. Future studies may also consider the effect of potential covariance between CH_4_, CO, or VOCs and other related pollutants (*e.g.*, CO_2_) on sensor calibration depending on nearby contributing sources.

Finally, this study also benefits from sensor evaluation in both laboratory and field settings to understand inter-sensor variability and to better correct for the sensor's cross-sensitivities and environmental dependencies. Laboratory calibrations can be useful to determine which predictors to monitor in the field. However, the conditions are typically unlike the true environmental conditions of deployment which involve various mixtures of pollutants and conditions not easily replicable in a controlled laboratory setting. Thus, while the laboratory chamber experiments presented in the study were helpful to inform the predictors that needed to be considered in the field calibration model, a lab-based correction based on sensor responses to various predictors in the lab was non-transferable to field co-located data (not shown). This is consistent with previous unpublished attempts to provide a lab-based calibration for the sensor by Eugster *et al.*^20^

We also note that only a sensor-specific calibration is presented in this study as proof of concept for applying a CO correction to improve sensor operation. Our inter-sensor comparison results suggest that the sensors exhibit varying magnitudes of response to environmental conditions and cross-sensitive pollutants. Thus, the predictor coefficients presented in this study are not directly transferable to other units. Co-locating the individual sensors with a reference instrument over a range of conditions comparable to the environment in which the sensor will ultimately be deployed is recommended.

## 4. Conclusion

Routine, continuous CH_4_ measurement at finer spatial scales to facilitate monitoring across complex urban settings will likely require deployments of low-cost sensors in tandem with other multi-platform measurement methods. This study examined the Figaro TGS2600 sensor for ambient CH_4_ monitoring across seasons in the mid-Atlantic climate and presented novel multivariate calibrations with CO using an onboard low-cost CO sensor for coincident corrections of CO interferences. After correcting for CO, temperature, AH, and adjusting for the hour of day, the sensor showed useful performance compared to the Picaro CRDS instrument at 1 h resolution. Deployments of these low-cost methane sensors thus require multipollutant monitoring packages with temperature, humidity, and CO measurements in the sensing region. The sensor's ability to capture CH_4_ trends at 1 h, with improved performance in high variability conditions, makes it a promising candidate to measure CH_4_ in complex urban environments with the potential to identify (and monitor) hotspots or areas of concern (*e.g.*, fenceline monitoring).

## Conflicts of interest

There are no conflicts to declare.

## Supplementary Material

EA-003-D2EA00100D-s001
