# Supplementary material for: Laboratory and field evaluation of a low-cost methane sensor and key environmental factors for sensor calibration
Source: Environ Sci Atmos. 2023 Feb 21;3(4):683–94. doi: 10.1039/d2ea00100d (PMC10100561; doi:10.1039/d2ea00100d)
Supplement: EA-003-D2EA00100D-s001 [file EA-003-D2EA00100D-s001.pdf]

Electronic Supplementary Material (ESI) for Environmental Science: Atmospheres.
This journal is © The Royal Society of Chemistry 2023

Supplemental Material

Laboratory and Field Evaluation of a Low-cost Methane Sensor and Key Environmental Factors for Sensor Calibration

Joyce J.Y. Lin, Colby Buehler, Abhirup Datta, Drew R. Gentner, Kirsten Koehler, Misti Levy Zamora*

Table of Contents

Supplemental Figure 1. Inter-sensor correlation matrix of 8 sensors co-located in lab room over 8 consecutive days.
Supplemental Figure 2. Inter-sensor comparison for 8 sensors co-located in a laboratory over 8 consecutive days at 1-h resolution.
Supplemental Figure 3. Sensor responses to A) CH_4, B) NO, C) NO_2, and D) CO_2 in calibration chamber experiments.
Supplemental Table 1. Summary of calibration models tested.
Supplemental Table 2a. Model fits for data at 1-hr resolution for both winter and summer seasons.
Supplemental Table 2b. Model fits for data at 1-hr resolution for the winter season
Supplemental Table 2c. Model fits for data at 1-hr resolution for the summer season
Supplemental Table 3. Model 5 R^2 by different calibration and validation period splits

Supplemental Figure 1. Inter-sensor correlation matrix of 8 sensors co-located in lab room over 8 consecutive days.

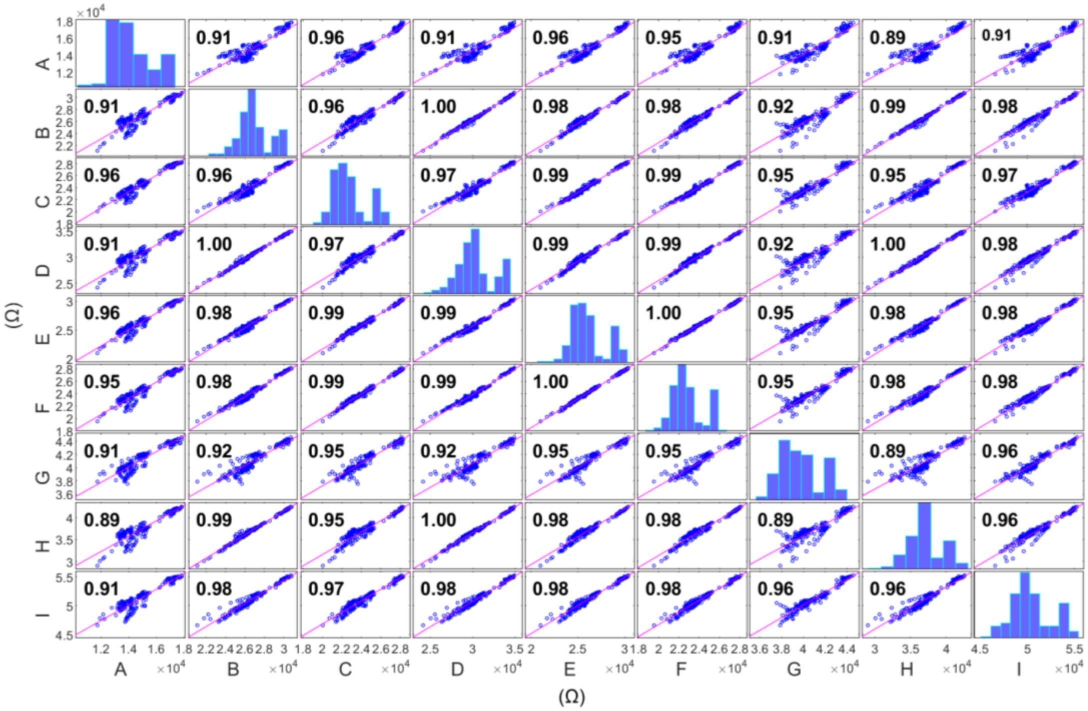

Supplemental Figure 2. Inter-sensor comparison for 8 sensors co-located in a laboratory over 8 consecutive days at 1-h resolution. This figure accompanies figure 1. in the main text and shows sensor values not normalized by their baseline resistances for comparison.

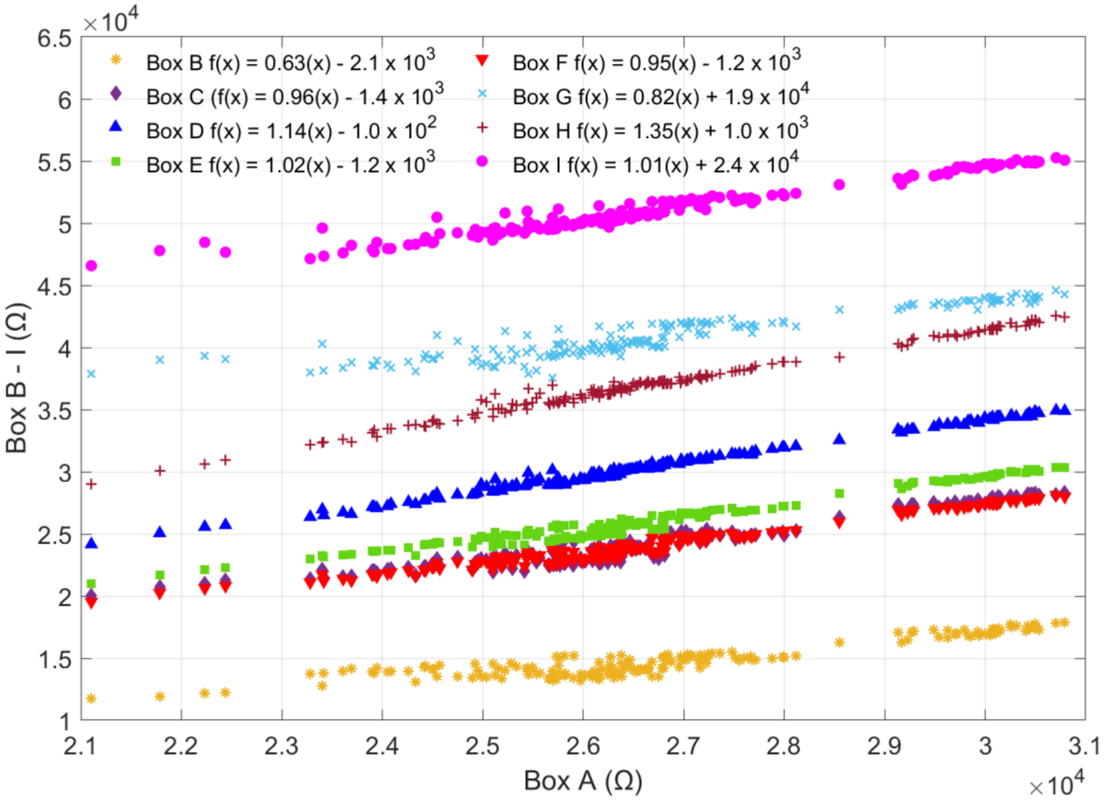

Supplemental Figure 3. Sensor responses to A) CH_4, B) NO, C) NO_2, and D) CO_2 in calibration chamber experiments. CH_4 is repeated from the main text but fit to the same sensor response scale as the other pollutants to highlight the sensor's limited responses to CO_2, NO, and NO_2 in the calibration chamber experiments. Ambient concentrations of CO_2, NO, and NO_2 exist on different scales than CH_4 concentrations, thus the slopes cannot be directly compared. However, median ambient concentrations of CO_2, NO, and NO_2 typically range from 420 – 490 ppm, 0 – 0.040 ppm, and 0 – 0.040 ppm, respectively, in the study area. Within these concentration ranges, the CH_4 sensor response is effectively negligible.

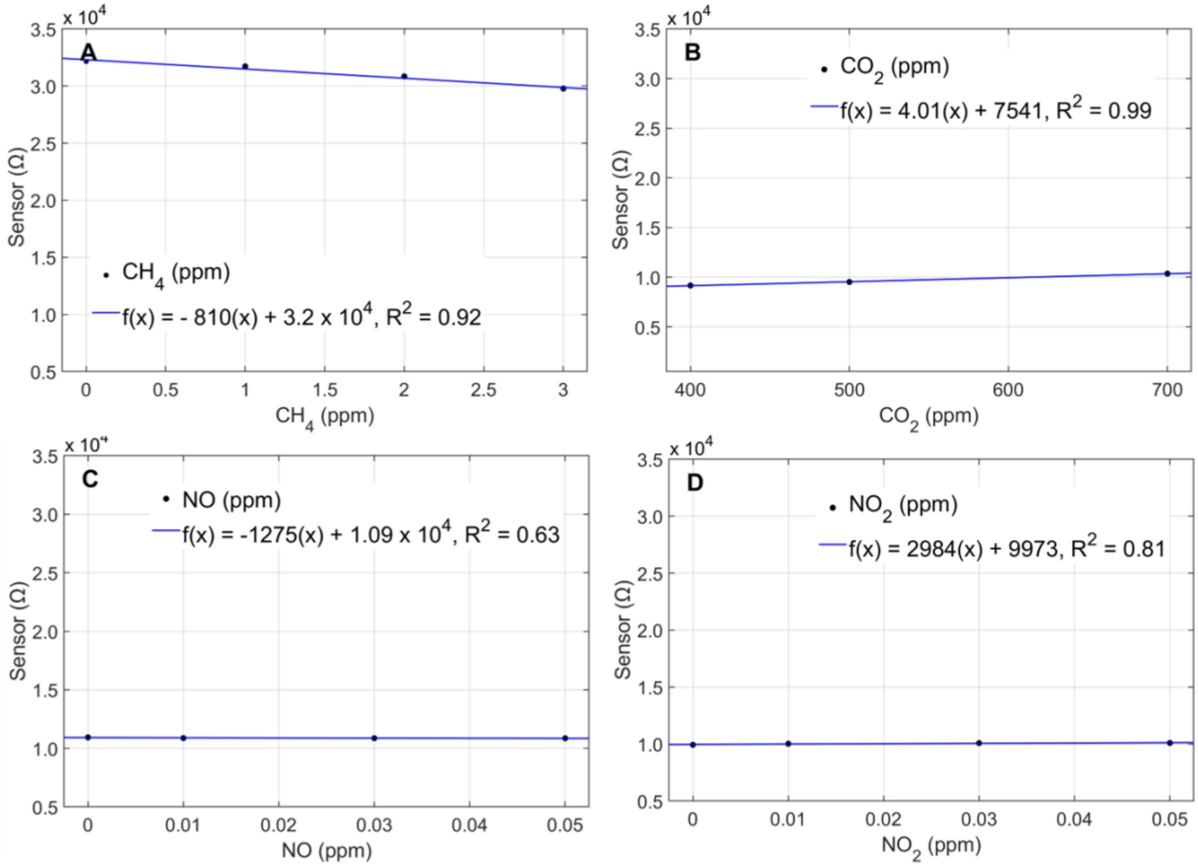

Supplemental Figure 4. CO sensor response to CO calibration in chamber experiments.

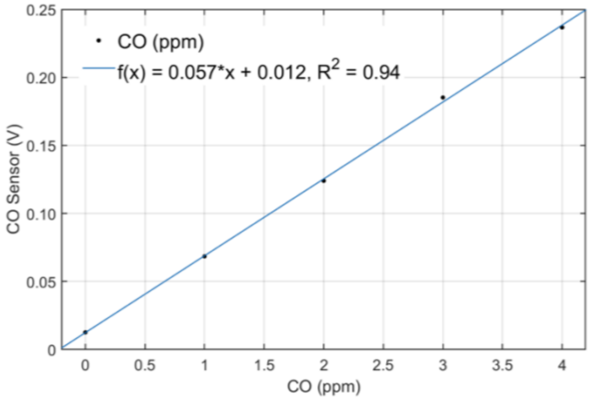

Supplemental Figure 5. Comparison of model with and without hour of day predictor (HOD). A) diurnal trend with HOD. B) Diurnal trend without HOD. C) Time series with HOD. D) Time series without HOD.

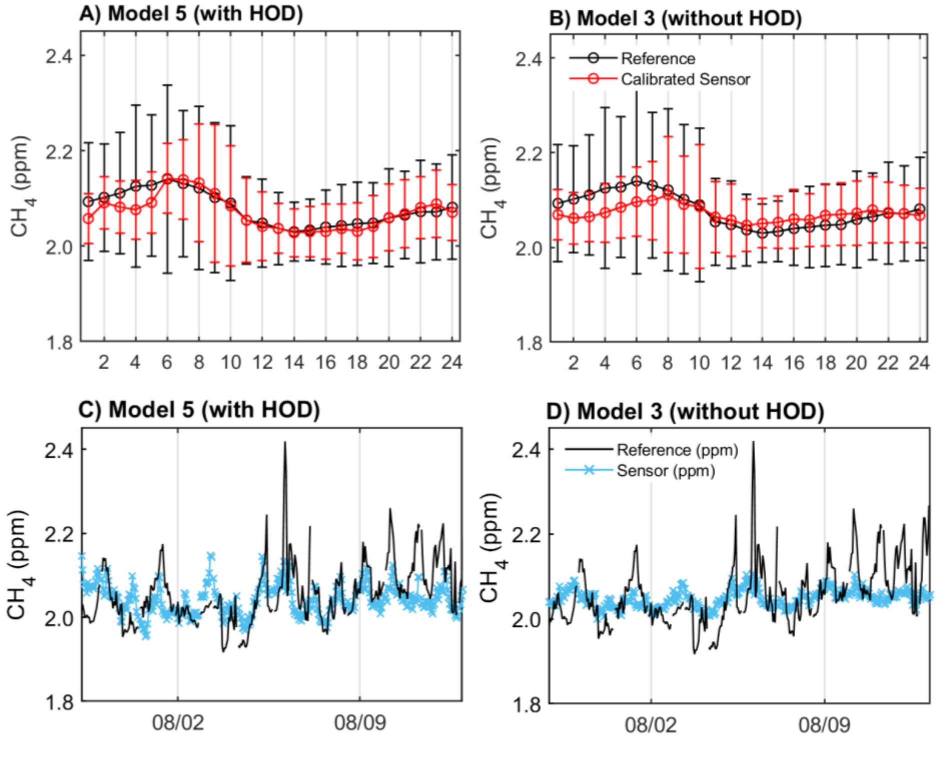

Supplemental Table 1. Summary of calibration models tested. R_s is sensor resistance. AH is absolute humidity. T is temperature. CO is corrected CO concentration from low-cost CO sensor. HOD is hour of day.

Model	Model Equation
0	$[CH_4] = \beta_0 + \beta_1 (R_s)$
1	$[CH_4] = \beta_0 + \beta_1 (R_s) + \beta_2 (AH)$
1a	$[CH_4] = \beta_0 + \beta_1 (R_s) + \beta_2 (AH)^{0.5}$
1b	$[CH_4] = \beta_0 + \beta_1 (R_s) + \beta_2 (AH)_{spline}$
**2**	**$[CH_4] = \beta_0 + \beta_1 (R_s) + \beta_2 (AH) + \beta_3 (T)$**
2a	$[CH_4] = \beta_0 + \beta_1 (R_s) + \beta_2 (AH)^{0.5} + \beta_3 (T)$
2b	$[CH_4] = \beta_0 + \beta_1 (R_s) + \beta_2 (AH)^{0.5} + \beta_3 (T)^2$
**3**	**$[CH_4] = \beta_0 + \beta_1 (R_s) + \beta_2 (AH) + \beta_3 (T) + \beta_4 (CO)$**
3a	$[CH_4] = \beta_0 + \beta_1 (R_s) + \beta_2 (AH)^{0.5} + \beta_3 (T)^2 + \beta_4 (CO)$
4	$[CH_4] = \beta_0 + \beta_1 (R_s) + \beta_2 (AH)^{0.5} + \beta_3 (T)^2 + \beta_4 (CO) + \beta_5(HOD)$
**5***	**$[CH_4] = \beta_0 + \beta_1 (R_s) + \beta_2 (AH) + \beta_3 (T) + \beta_4 (CO) + \beta_5(HOD)$**
6	$[CH_4] = \beta_0 + \beta_1 (R_s) + \beta_2 (AH) + \beta_3 (T) + \beta_4 (CO) + \beta_5(HOD) + \beta_6(Rs*AH)$
7	$[CH_4] = \beta_0 + \beta_1 (R_s) + \beta_2 (AH) + \beta_3 (T) + \beta_4 (CO) + \beta_5(HOD) + \beta_6(Rs*T)$
8	$[CH_4] = \beta_0 + \beta_1 (R_s) + \beta_2 (AH) + \beta_3 (T) + \beta_4 (CO) + \beta_5(HOD) + \beta_6(AH*T)$

Bolded models are included in the main text. * Indicates best fitting model chosen for sensor calibration

Supplemental Table 2a. Model fits for data at 1-hr resolution for both winter and summer seasons.

	Calibration			Validation		
Model	Percent Bias (%)	RMSE (ppm)	R^2	Percent Bias (%)	RMSE (ppm)	R^2
**0**	**3.59**	**0.12**	**0.00**	**3.52**	**0.13**	**0.01**
1	3.15	0.10	0.21	3.71	0.11	0.19
1a	3.11	0.10	0.21	3.97	0.11	0.21
1b	3.08	0.09	0.23	4.08	0.11	0.18
**2**	**3.11**	**0.10**	**0.22**	**3.73**	**0.11**	**0.21**
2a	3.10	0.10	0.21	3.96	0.11	0.21
2b	3.08	0.10	0.22	3.94	0.11	0.22
**3**	**2.68**	**0.09**	**0.37**	**2.82**	**0.08**	**0.52**
3a	2.69	0.09	0.36	2.83	0.08	0.53
4	2.61	0.08	0.41	2.76	0.08	0.54
**5***	**2.61**	**0.08**	**0.43**	**2.69**	**0.08**	**0.55**
6	2.61	0.08	0.44	2.49	0.08	0.57
7	2.59	0.08	0.44	2.55	0.08	0.58
8	2.58	0.08	0.44	2.46	0.08	0.59

Bolded models are included in the main text. * Indicates best fitting model chosen for sensor calibration

Supplemental Table 2b. Model fits for data at 1-hr resolution for the winter season

	Calibration			Validation		
Model	Percent Bias (%)	RMSE (ppm)	R^2	Percent Bias (%)	RMSE (ppm)	R^2
**0**	**3.82**	**0.13**	**0.17**	**3.90**	**0.14**	**0.23**
1	3.66	0.12	0.29	5.28	0.14	0.38
1a	3.61	0.12	0.29	5.69	0.15	0.44
1b	3.54	0.12	0.34	7.53	0.18	0.49
**2**	**3.71**	**0.12**	**0.31**	**5.39**	**0.14**	**0.34**
2a	3.56	0.12	0.36	6.36	0.15	0.39
2b	3.55	0.12	0.35	6.36	0.15	0.42
**3**	**3.04**	**0.10**	**0.42**	**3.09**	**0.09**	**0.65**
3a	3.03	0.10	0.42	3.35	0.10	0.66
4	3.08	0.10	0.47	3.43	0.10	0.65
**5***	**3.08**	**0.10**	**0.47**	**3.12**	**0.09**	**0.65**
6	3.13	0.10	0.45	3.36	0.10	0.66
7	2.83	0.09	0.51	4.08	0.11	0.56
8	3.08	0.10	0.47	3.06	0.09	0.64

Bolded models are included in the main text. * Indicates best fitting model chosen for sensor calibration

Supplemental Table 2c. Model fits for data at 1-hr resolution for the summer season

	Calibration			Validation		
Model	Percent Bias (%)	RMSE (ppm)	R^2	Percent Bias (%)	RMSE (ppm)	R^2
**0**	**2.16**	**0.06**	**0.03**	**2.88**	**0.08**	**0.01**
1	2.17	0.06	0.05	2.87	0.08	0.04
1a	2.17	0.06	0.05	2.87	0.08	0.05
1b	2.11	0.06	0.10	2.87	0.08	0.14
**2**	**2.17**	**0.06**	**0.07**	**2.63**	**0.07**	**0.19**
2a	2.17	0.06	0.07	2.63	0.07	0.20
2b	2.17	0.06	0.07	2.64	0.07	0.20
**3**	**2.04**	**0.06**	**0.19**	**2.24**	**0.06**	**0.33**
3a	2.04	0.06	0.19	2.25	0.06	0.33
4	1.84	0.05	0.40	2.99	0.08	0.13
**5***	**1.83**	**0.05**	**0.40**	**3.01**	**0.08**	**0.12**
6	1.83	0.05	0.40	2.34	0.07	0.33
7	1.83	0.05	0.40	2.92	0.08	0.14
8	1.82	0.05	0.41	2.86	0.08	0.15

Bolded models are included in the main text. * Indicates best fitting model chosen for sensor calibration

Supplemental Table 3. Model 5 R^2 by different calibration and validation period splits

Calibration/validation period split	Calibration R^2	Validation R^2
**1:3**<br>First 2.5 weeks of winter and summer season for calibration, remaining data for validation	0.43	0.55
**5:4**<br>First 5 weeks of winter and summer for calibration, remaining data for validation	0.55	0.57
**1:3 reverse**<br>Last 2.5 weeks of winter and summer for calibration, remaining data for validation	0.64	0.34
**5:4 reverse**<br>Last 5 weeks of winter and summer for calibration, remaining data for validation	0.66	0.46
